# Development and validation of a prognostic score for TIPS placement in patients with viral hepatitis cirrhosis-related portal hypertension: a multi-center retrospective study

**DOI:** 10.3389/fmed.2024.1456758

**Published:** 2024-09-30

**Authors:** Zenglong Que, Mingsong Wu, Shujie Lai, Lei Wang, Zhiyong Mu, Jinhui Yang, Wei Xiong, Hong Hu, Aimin Liu, Xuan An, Haodong Yu, Qin Cao, Yanmei Zhang, Wenwen Luo, Jun Wang, Dongfeng Chen, Fuquan Liu, Dazhi Zhang, Liangzhi Wen

**Affiliations:** ^1^Department of Gastroenterology, Chongqing Key Laboratory of Digestive Malignancies, Daping Hospital, Army Medical University (Third Military Medical University), Chongqing, China; ^2^Department of Infectious Diseases, The 960 Hospital of PLA, Jinan, China; ^3^Department of Interventional Therapy, Beijing Shijitan Hospital, Capital Medical University, Beijing, China; ^4^Department of Hepatology, The Second Affiliated Hospital of Kunming Medical University, Kunming, China; ^5^Department of Gastroenterology, The Third Affiliated Hospital of Chongqing Medical University, Chongqing, China; ^6^Department of Interventional Radiology, Nanchong Hospital Affiliated to North Sichuan Medical College, Nanchong, China; ^7^Department of Gastroenterology, Fuling Hospital Affiliated to Chongqing University, Chongqing, China; ^8^Department of Hepatology, Three Gorges Hospital Affiliated to Chongqing University, Chongqing, China; ^9^Department of Gastroenterology, Qianjiang Hospital, Chongqing, China; ^10^Department of Infection, The Second Affiliated Hospital of Chongqing Medical University, Chongqing, China

**Keywords:** viral hepatitis, cirrhosis, portal hypertension, prognosis, mortality, model

## Abstract

**Introduction:**

There is no established scoring model focused on viral hepatitis patients to predict the prognosis after transjugular intrahepatic portosystemic shunt (TIPS). We aimed to develop and validate a novel model based on the largest cohort for better prediction of both short-term (1 year) and long-term (3 years) postoperative prognoses after TIPS in viral hepatitis cirrhosis-related portal hypertension patients.

**Methods:**

A total of 925 viral hepatitis cirrhosis-related portal hypertension patients who underwent TIPS from nine hospitals were divided into the training and external validation cohorts. A novel Viral-associated Index of Post-TIPS score (VIPs) model was developed after performing Cox regression analysis. The VIPs model was compared to five previous models, namely, Child–Pugh, MELD, ALBI, CCG, and FIPS. Furthermore, X-tile software was used to stratify patients into low-, medium-, and high-risk groups.

**Results:**

The VIPs model included age, ascites, albumin, prothrombin time, total bilirubin, and sodium for post-TIPS prognosis prediction. The model demonstrated satisfying predictive efficiency in both discrimination and calibration, with an area under the curve of 0.781/0.774 (1 year/3 years) in the training cohort and 0.771/0.775 (1 year/3 years) in the external validation cohort, respectively.

**Discussion:**

We first developed and externally validated a novel VIPs model for better prediction of both short-term and long-term postoperative prognoses after TIPS in Chinese patients with viral hepatitis cirrhosis-related portal hypertension.

## 1 Introduction

Transjugular intrahepatic portosystemic shunt (TIPS) is a recommended microinvasive treatment for complications caused by cirrhosis-related portal hypertension, such as esophagogastric variceal bleeding and refractory ascites (1–4). However, TIPS might increase the incidence rate of liver cirrhosis complications including hepatic encephalopathy and acute liver failure, which result in worse prognosis and quality of life (5, 6). Therefore, patient selection is particularly important before TIPS implantation.

The non-invasive method is an effective and promising way to predict the prognosis after TIPS. To date, several non-invasive clinical models were used for TIPS prognosis prediction, such as Child–Turcotte–Pugh (CTP) score (7), Model for End-Stage Liver Disease (MELD) score (8), albumin–bilirubin (ALBI) score (9), CLIF Consortium Acute Decompensation score (CLIF-C AD) (10), and Freiburg index of post-TIPS survival (FIPS) score (11). However, the models including CTP, MELD, ALBI, and CLIF-C AD were not constructed based on the TIPS population, and they were commonly used to predict short-term mortality rates in patients with end-stage liver disease or hepatocellular carcinoma. The FIPS score was the latest model for TIPS prognosis prediction based on the TIPS population, while its predictive ability remains to be further explored, especially in the Chinese cohort. FIPS, similar to models mentioned above, was based on Western cirrhosis cohorts and might not be suitable for the Chinese population, because cirrhosis in Western countries was most commonly caused by alcohol-related liver disease and non-alcoholic fatty liver disease (NAFLD), while cirrhosis in China is most commonly caused by viral hepatitis (HBV and HCV). Furthermore, our previous research and Han’s cohort showed that the FIPS score might not be better than the CTP score in the Chinese population (12).

Viral hepatitis (HBV and HCV) was the main cause of cirrhosis in the Chinese population, accounting for approximately 79% (13). However, there was a lack of an appropriate scoring model for TIPS postoperative survival prediction in those patients. The purpose of this study was to first develop and externally validate a novel model for better prediction of both short-term (1 year) and long-term (3 years) postoperative prognoses after TIPS in patients with viral hepatitis cirrhosis-related portal hypertension. Moreover, our model was developed in the southwest (eight centers) and externally validated in the northeastern (one center) population of China, which was more representative and showed stable predictive ability in Chinese cohorts.

## 2 Materials and methods

### 2.1 Study population

The ethical committees of all participating hospitals approved the research protocol. All research studies were conducted in accordance with both the Declaration of Helsinki. Written consent was given by all patients. This study was conducted among patients with viral hepatitis cirrhosis-related portal hypertension who underwent TIPS placement. A retrospective analysis of clinical data was conducted among patients who underwent TIPS placement in nine tertiary hospitals from May 2011 to September 2022. The follow-up period ended on 30 September 2023 or until the patient’s death. The patients from Beijing Shijitan Hospital who met the criteria were included in the validation cohort, and patients from the remaining eight centers were included in the training cohort for model construction. All patients received oral antiviral therapy, and follow-up revealed that HBV DNA levels or HCV RNA levels were below the detection limit. This article was written according to the TRIPOD guideline (14). The inclusion criteria for this study were as follows: (1) patients with viral hepatitis-related cirrhosis (based on clinical features, laboratory and imaging tests, or liver biopsy) who underwent TIPS placement and (2) patients aged between 18 and 80 years old. The exclusion criteria were as follows: (1) patients who had liver cirrhosis due to other causes; (2) patients who had previously received devascularization or portosystemic shunts; (3) patients who had hepatocellular carcinoma or other malignant tumors; (4) patients who had non-cirrhotic portal hypertension; and (5) patients who had severe organ dysfunction such as congestive heart failure, severe valvular heart insufficiency, and a creatinine level of >442 μmol/L.

### 2.2 Outcomes

The main endpoint of the study was all-cause mortality during the follow-up period of 1 or 3 years after TIPS placement.

### 2.3 Predictive factors and data collection

Since our goal was to develop a score that predicts postoperative survival based on preoperative clinical data, the predictive factors were limited to variables collected within 3 days prior to TIPS placement. Specific information included basic patient information, clinical characteristics, and laboratory tests, as shown in Table 1.

**Table 1:** Baseline characteristics of the study patients in the training cohort and the validation cohort.

Variables	Training cohort, *n* = 709	Validation cohort, *n* = 216	*p*
Age (years)	49 ± 10	52 ± 11	0.001
Sex, *n* (%)			0.549
Men	534 [75.3]	167 [77.3]	
Women	175 [24.7]	49 [22.7]	
Indications for TIPS, *n* (%)			0.750
Variceal bleeding	662 [93.4]	203 [94]	
Ascites	47 [6.6]	13 [6]	
TIPS stent, *n* (%)			0.000
Covered stent	623 [87.9]	214 [99.1]	
Bare stent	86 [12.1]	2 [0.9]	
Ascites, *n* (%)			0.075
None	275 [38.8]	85 [39.4]	
Moderate	270 [38.1]	53 [24.5]	
Massive	164 [23.1]	78 [36.1]	
WBC (10^9^/L)	3.16 (2.14–4.79)	2.97 (1.77–4.2)	0.017
Hb (g/L)	85 (71–103)	92 (79–115)	0.000
PLT (10^9^/L)	54 (39–80)	75 (49–121)	0.000
ALT (U/L)	25 (17–37)	20 (15–27)	0.000
AST(U/L)	32 (24–44)	29 (21–35)	0.000
Alb (g/L)	34.7 ± 5.9	36 ± 5	0.001
INR	1.29 (1.17–1.46)	1.31 (1.19–1.43)	0.977
TBil (μmol/L)	20.3 (13.7–29.8)	21.5 (15.8–31.8)	0.015
Scr (μmol/L)	69 (58.1–81.3)	64 (55–76)	0.002
Na (mmol/L)	139.4 (137.2–141.0)	140 (138–142)	0.001
Child–Pugh score	7 (6–8)	7 (6–8)	0.689
MELD score	11 (9–14)	11 (9–13)	0.084
ALBI score	−2.08 ± 0.56	−2.15 ± 0.46	0.047
FIPS score	−1.03 (−1.47–−0.60)	−1.11 (−1.57–−0.51)	0.508
CCG-AVB-TEP1 score	−0.67 (−0.93–−0.36)	−0.71 (−0.94–−0.37)	0.438
Follow-up time(d)	1,027 (605–1,522)	1,268 (1137–1,494)	0.000

Mean ± standard deviation for normally distributed variables, medians and interquartile ranges for non-normally distributed variables, and numbers [proportions, %] for categorical variables.

Hb, hemoglobin; PLT, platelet; ALT, alanine aminotransferase; AST, aspartate aminotransferase; Alb, albumin; INR, international normalized ratio; TBil, total bilirubin; Scr, serum creatinine; MELD, Model for End-Stage Liver Disease; ALBI, albumin–bilirubin; FIPS, Freiburg index of post-TIPS survival; CCG-AVB-TEP1, Chinese Collaboration Group on the Acute Variceal Bleeding score and Predicting the Treatment Effect.

### 2.4 Sample size calculation

The sample size was calculated using the method proposed by Richard D. Riley et al. (15). To develop a multivariate clinical prediction model, seven predictor parameters were included in the final model, with an adjusted R-squared of 0.1, shrinkage of 10%, and the 1-year mortality rate (outcome event) of 13%. Based on these assumptions, the minimum sample size required for model development is 595 patients with 78 outcome events. In this study, a total of 925 patients were finally enrolled, including 112 patients with outcome events.

### 2.5 Statistical analysis

All statistical analyses were performed using SPSS (version 26.0) or R (version 4.3.1), with the following packages: pmsampsize, car, survival, plyr, forestplot, ggplot2, survminer, rms, pROC, ggDCA, nomogramEx, nomogramFormula, and MASS. Statistical significance was set at *p* < 0.05 (two-sided). Normally distributed variables are expressed as the mean ± standard deviation and were compared using an independent-samples *t*-test. Non-normally distributed variables are expressed as medians and interquartile ranges and were compared using the Mann–Whitney rank-sum test. Categorical variables are expressed as numbers (proportions, %) and were compared using the *χ*2 test.

### 2.6 Prediction of the survival probabilities in patients under TIPS placement

Univariate Cox regression analysis was used to identify factors significantly associated with survival after TIPS placement, and variables with a *p*-value of <0.05 in the univariate analysis were included in subsequent multivariate analysis. Multivariate analysis used backward stepwise regression to select the prediction model with the minimum Akaike information criterion. In the final model, the prognostic score for each individual was calculated, and a nomogram was developed to calculate the survival prediction probabilities for 1 and 3 years.

The performance of the model was evaluated and validated through Harrell’s C-statistic (C-index) (16) and calibration plot (17). Decision curve analysis (DCA) was used to assess the clinical utility of the model, which quantifies the net benefit at different threshold probabilities (18). The performance of the new model was compared to the Child–Pugh score, MELD score, ALBI score, FIPS score, and the Chinese Collaboration Group on the Acute Variceal Bleeding and Predicting the Treatment Effect (CCG-AVB-TEP1) model (19). The bootstrap method was used with 1,000 iterations to provide an unbiased estimate of the model’s performance as the C-index. For external validation, the prognostic score for each individual in the validation cohort was calculated using the formula developed in the training cohort. The external validity was assessed by calculating the C-index and calibration plot and compared with other scoring models. X-tile software (20) was used to determine the optimal cutoff value for prognostic scoring, and a log-rank test was performed to assess whether there were differences among the risk groups and to distinguish the low-risk, medium-risk, and high-risk populations. The performance of the prognostic score in subgroups was assessed according to the etiology [hepatitis B virus (HBV) vs. hepatitis C virus (HCV)].

### 2.7 Score calculation

The Child–Pugh score included five parameters: total bilirubin, albumin, prothrombin time, ascites, and hepatic encephalopathy. The final score was obtained by summing up the scores of each parameter. The formula for the MELD score is 3.8 × ln [(TBIL, μmol/L) ÷ 17.1] + 11.2 × ln (INR) +9.6 × ln [(creatinine, μmol/L) ÷ 88.4] + 6.4 × etiology (cholestasis or alcohol 0, others 1). The formula for the ALBI score is 0.66 × lg (TBIL, μmol/L) − 0.085 × (albumin, g/L). The formula for the FIPS score is 1.43 × lg (TBIL, μmol/L) − 1.71 × 1/ (creatinine, mg/dL) + 0.02× (age, years) − 0.02 × (albumin, g/L). The formula for the CCG-AVB-TEP1 score is 10 × [0.0289 × (age, years) − 0.0525 × (albumin, g/L) + 0.3334 × Ln (bilirubin, mg/dl) + 1.7631 × Ln (INR) + 0.3373 × Ln (WBC, 10^9^/L) + 0.4509 × Ln (creatinine, mg/dl) − 0.0294 × (sodium, mmol/L) + 8] × 0.056838484–3.1231513.

## 3 Results

### 3.1 Baseline characteristics of patients and outcomes

**Figure 1:**
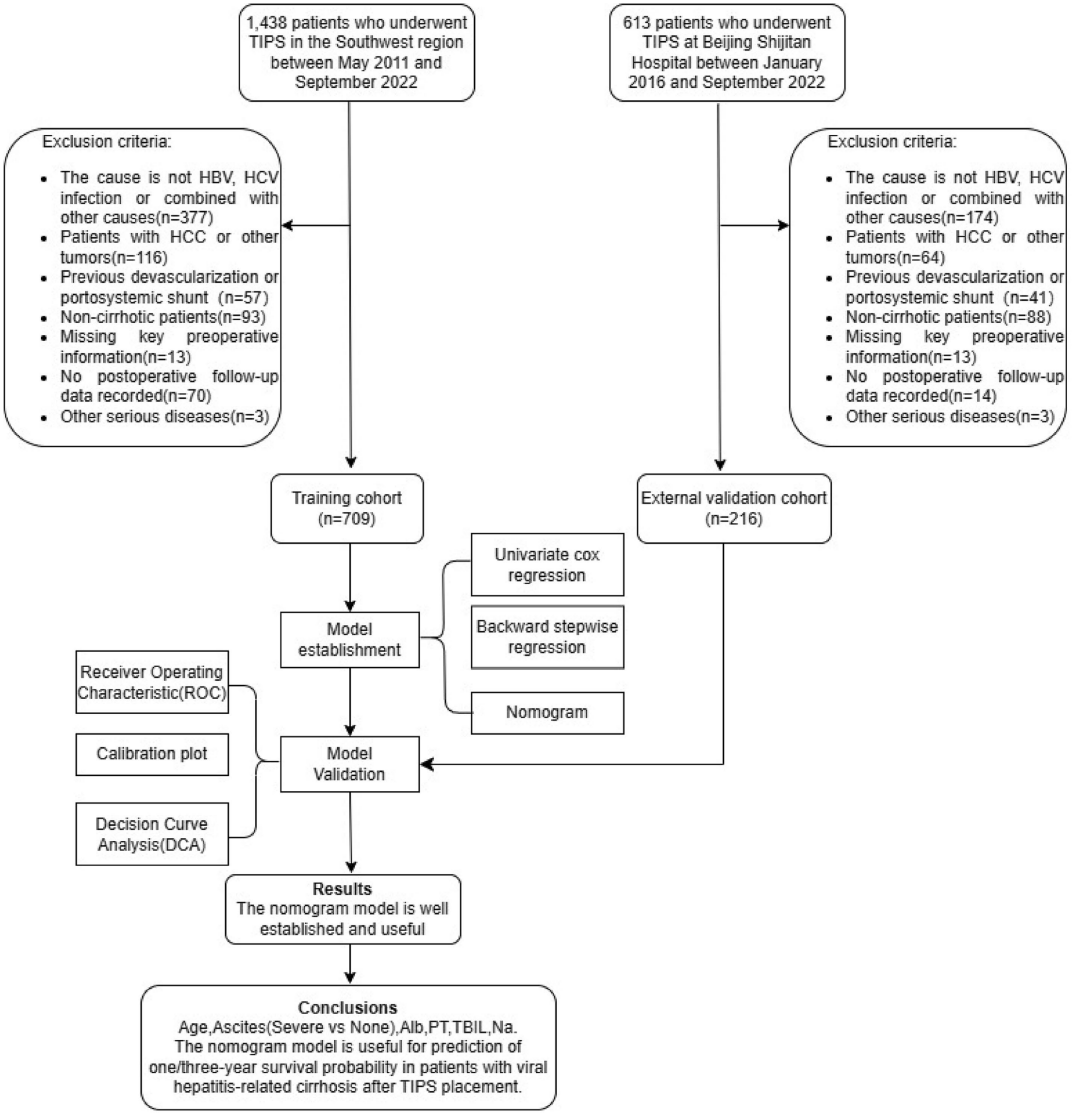
Flowchart showing the study design and patient disposition.

A total of 2051 patients underwent TIPS placement at the centers. To develop a survival prediction model for TIPS placement, 1,126 patients who did not meet the inclusion criteria were excluded, and 925 patients were included in the subsequent model development and validation. The training cohort included 709 patients, while the validation cohort included 216 patients (Figure 1). The baseline characteristics of the two cohorts are shown in Table 1.

### 3.2 Model development

**Figure 2:**
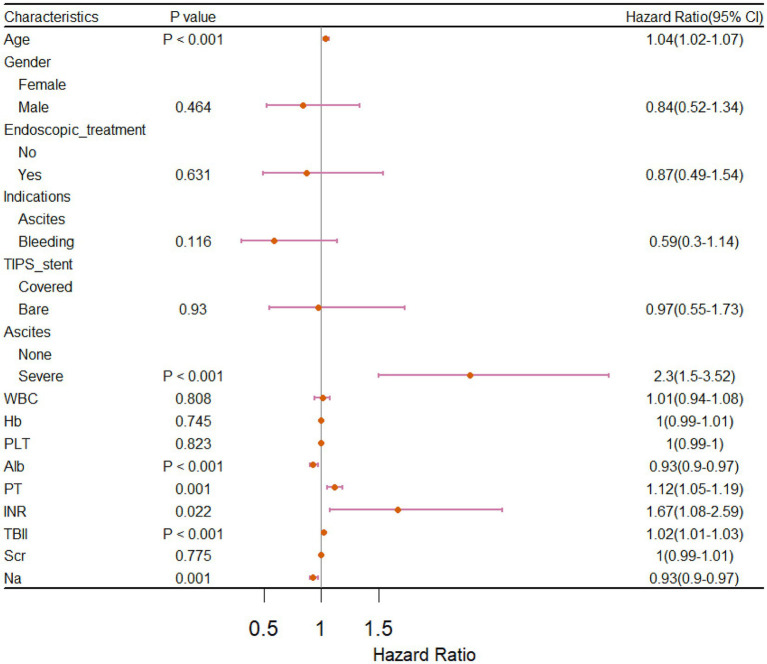
Forest plot of univariate Cox regression analysis for prognostic factors in patients who received TIPS.

The univariate Cox regression analysis of the training cohort showed that age, ascites, albumin, prothrombin time (PT), international normalized ratio (INR), total bilirubin (TBIL), and sodium (*p* < 0.05) were important risk factors for survival after TIPS placement (Figure 2). A backward stepwise regression method was used to develop a model with the selected variables, and the final multivariate model included age, ascites, albumin, PT, TBIL, and sodium (Na). Based on the final model, a risk score was developed and named the Viral-associated Index of Post-TIPS score (VIPs). The calculation formula for the risk score is as follows:

VIPs = 1.19 × age+9.01× ascites +1.9 × PT + 0.5 × TBIl − 1.12 × Alb − 1.09 × Na + 181.62[age, years; PT, s; TBIL, μmol/L; Alb, g/L; Na, mmol/L; ascites: none = 0, severe (moderate or large ascites with moderately symmetrical abdominal distention or have significant abdominal distension) = 1].

**Figure 3:**
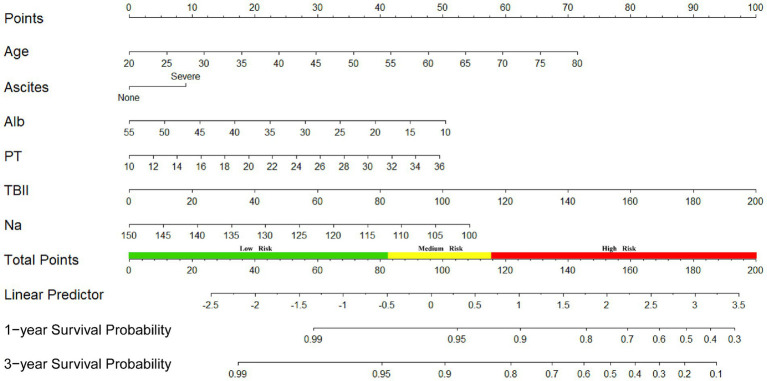
Nomogram to predict survival after TIPS.

We also developed a nomogram based on the final model to estimate the survival prediction probabilities for 1 and 3 years (Figure 3).

### 3.3 Model performance evaluation and internal validation

**Figure 4:**
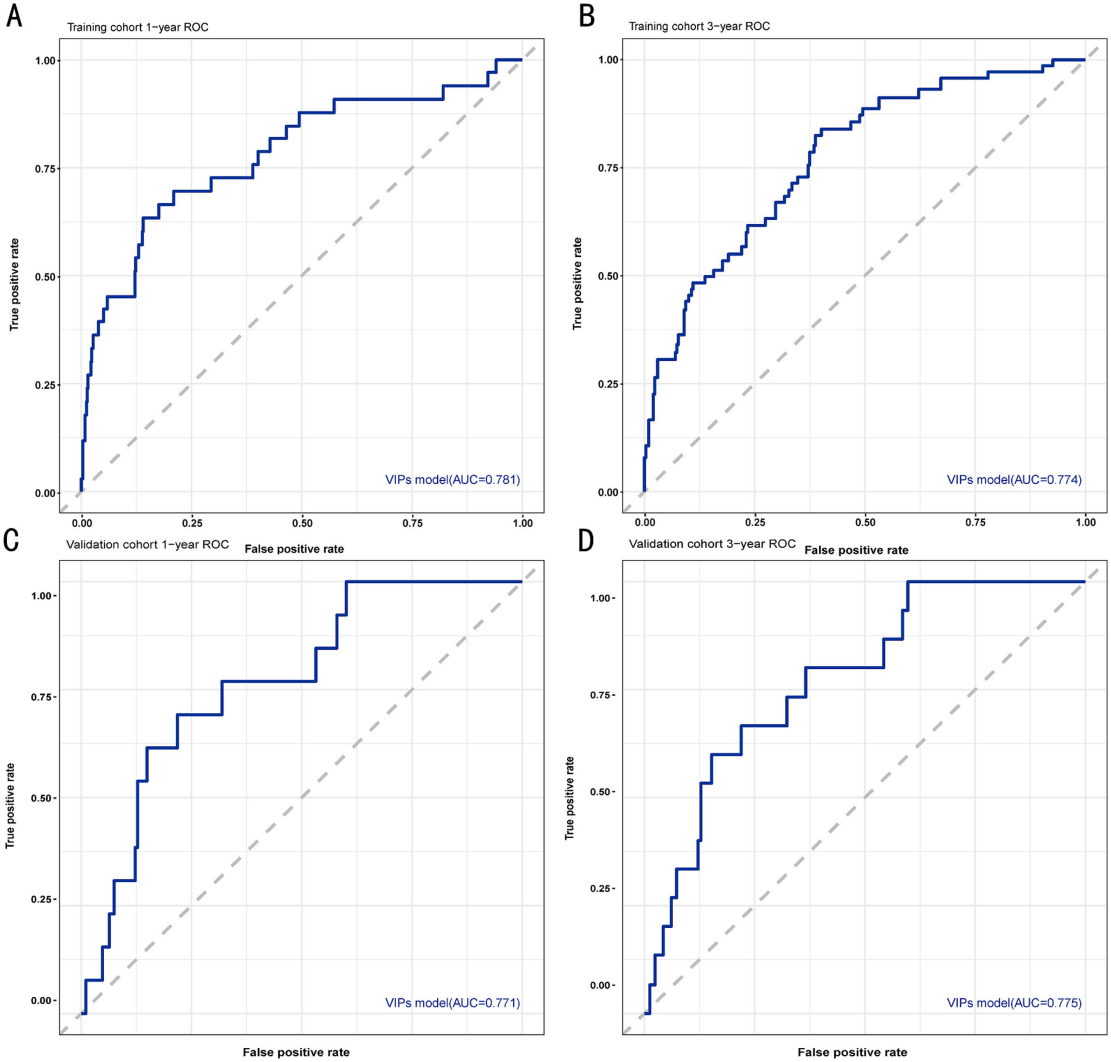
ROC curves for 1- and 3-year survival prediction of the VIP score model. **(A)** ROC curve for 1-year survival prediction using the training cohort. **(B)** ROC curve for 3-year survival prediction using the training cohort. **(C)** ROC curve for 1-year survival prediction using the validation cohort. **(D)** ROC curve for 3-year survival prediction using the validation cohort. The VIP score model showed excellent predictive value.

**Figure 5:**
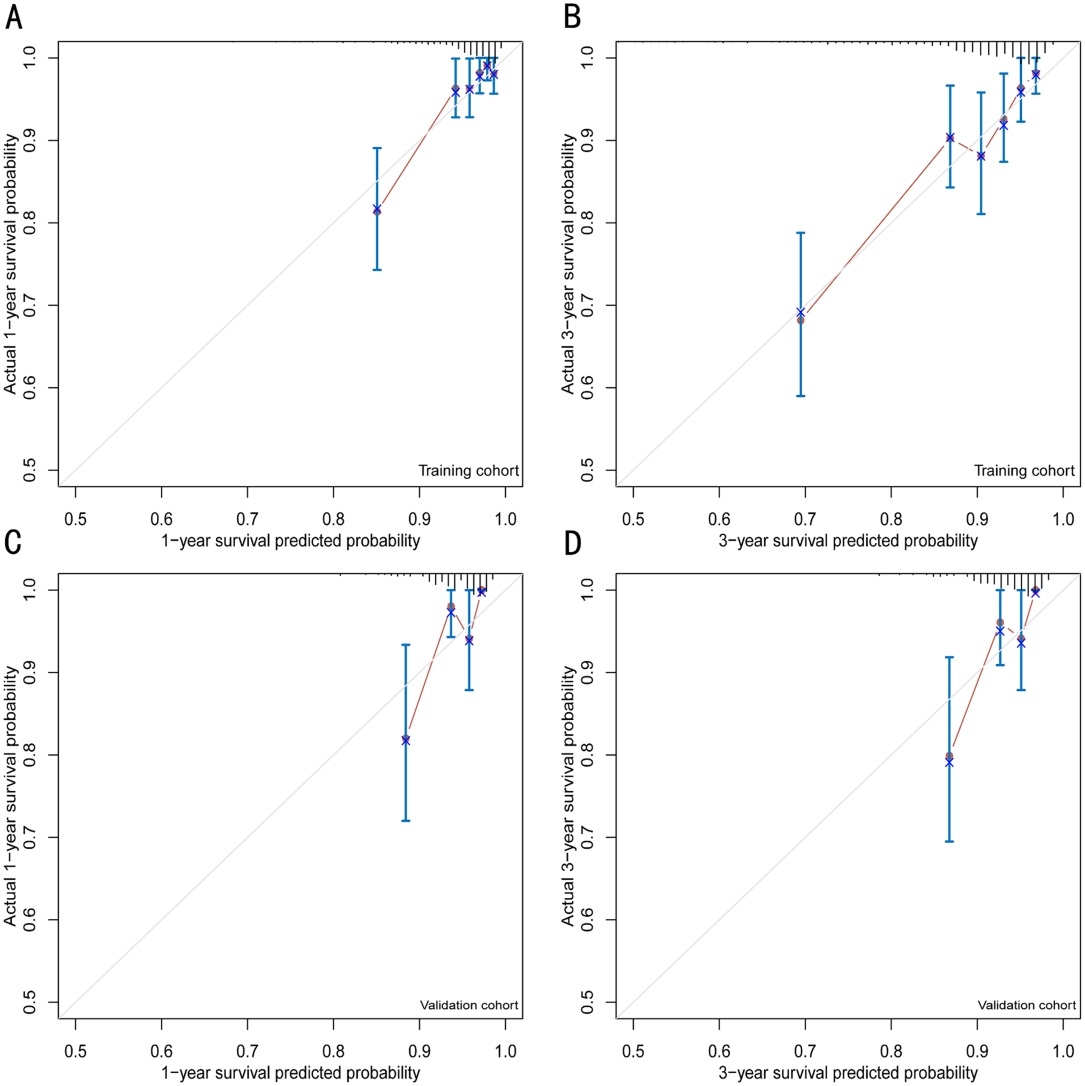
Calibration plots for 1- and 3-year survival prediction of the VIP score model. **(A)** Calibration plots for internal validation of the 1-year survival prediction using the training cohort. **(B)** Calibration plots for internal validation of the 3-year survival prediction using the training cohort. **(C)** Calibration plots for external validation of the 1-year survival prediction using the validation cohort. **(D)** Calibration plots for external validation of the 3-year survival prediction using the validation cohort.

**Figure 6:**
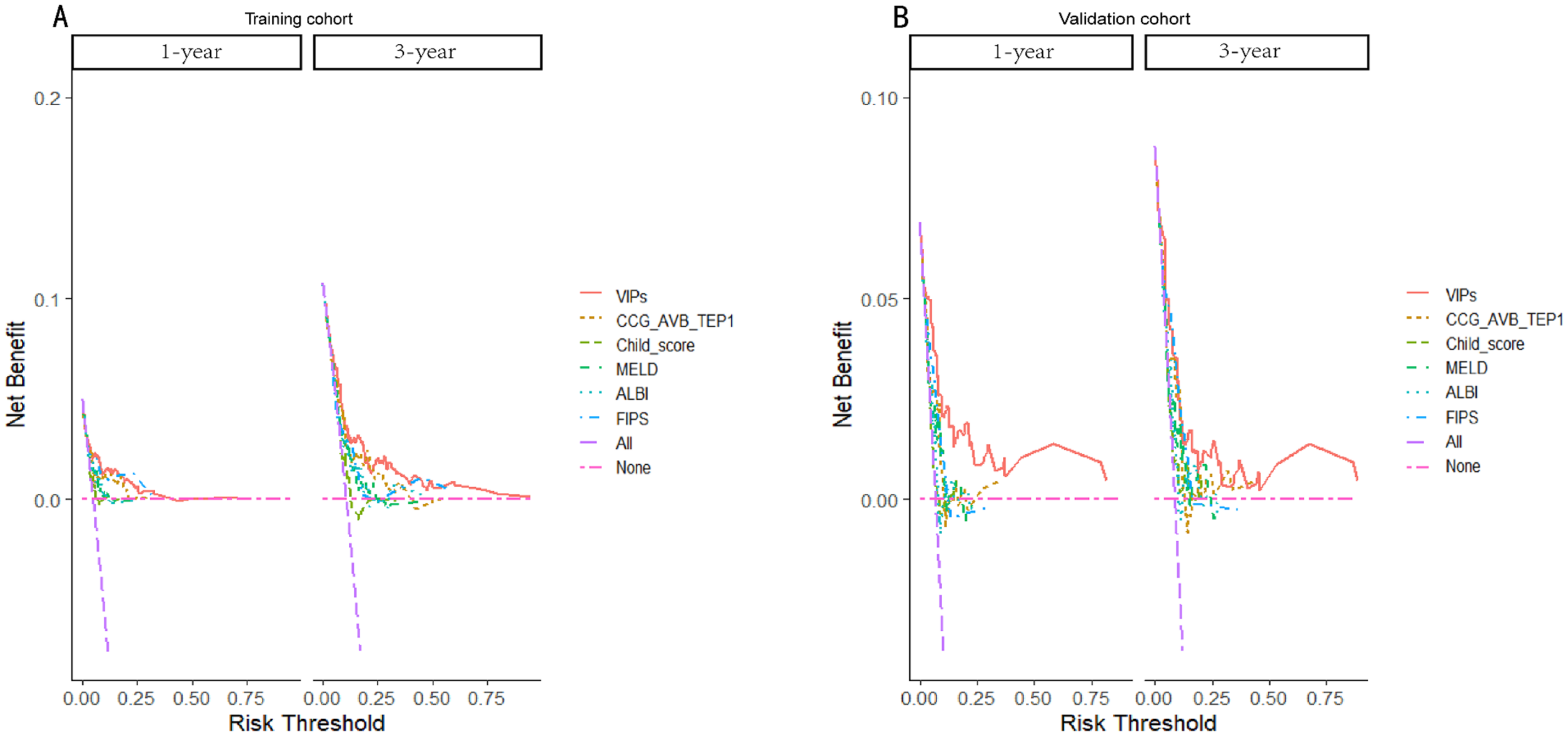
Decision curves of the VIP score model and other score prediction models for 1- and 3-year survival prediction after TIPS. **(A)** Decision curves for 1- and 3-year survival prediction using the training cohort. **(B)** Decision curves for 1- and 3-year survival prediction using the validation cohort.

The discrimination of the VIP score was evaluated by calculating the area under the receiver operating characteristic (ROC) curve. The area under the curve (AUC) of the VIP score for 1- and 3-year survival prediction in the training cohort was 0.781 and 0.774, respectively (Figures 4A,B). The calibration plot showed that the VIP score was well-calibrated at 1-year and 3-year survival (Figures 5A,B). DCA of the VIP score demonstrated a higher net benefit than the other score (Figure 6A). We performed internal validation using a bootstrap method with 1,000 iterations to provide an unbiased estimate of the VIP score performance as the C-index. The results showed that the VIP score for survival in the training cohort was 0.733, which was significantly better than the C-indices of the Child–Pugh score (0.672; *p* < 0.001), the MELD score (0.601, *p* < 0.001), the ALBI score (0.658, *p* < 0.001), the CCG-AVB-TEP1 score (0.701, *p* < 0.001), and the FIPS score (0.652, *p* < 0.001) (Table 2).

**Table 2:** The C-index of the VIP score model compared to the Child–Pugh, MELD, ALBI, FIPS, and CCG-AVB-TEP1 score models.

Model	Training cohort, C-index [95% CI]	*p*	Validation cohort, C-index [95% CI]	*p*
VIPs	0.733 [0.665–0.788]	–	0.869 [0.795–0.918]	–
Child–Pugh	0.672 [0.603–0.740]	<0.001	0.718 [0.640–0.796]	<0.001
MELD	0.601 [0.534–0.664]	<0.001	0.564 [0.439–0.689]	<0.001
ALBI	0.658 [0.593–0.723]	<0.001	0.641 [0.523–0.759]	<0.001
FIPS	0.652 [0.584–0.724]	<0.001	0.632 [0.522–0.742]	<0.001
CCG-AVB-TEP1	0.701 [0.640–0.765]	<0.001	0.690 [0.586–0.794]	<0.001

VIPs, Viral-associated Index of Post-TIPS score; CI, confidence interval; MELD, Model for End-Stage Liver Disease; ALBI, albumin–bilirubin; FIPS, Freiburg index of post-TIPS survival; CCG-AVB-TEP1, Chinese Collaboration Group on the Acute Variceal Bleeding score and Predicting the Treatment Effect.

### 3.4 External validation of the VIP score and subgroup analysis

**Figure 7:**
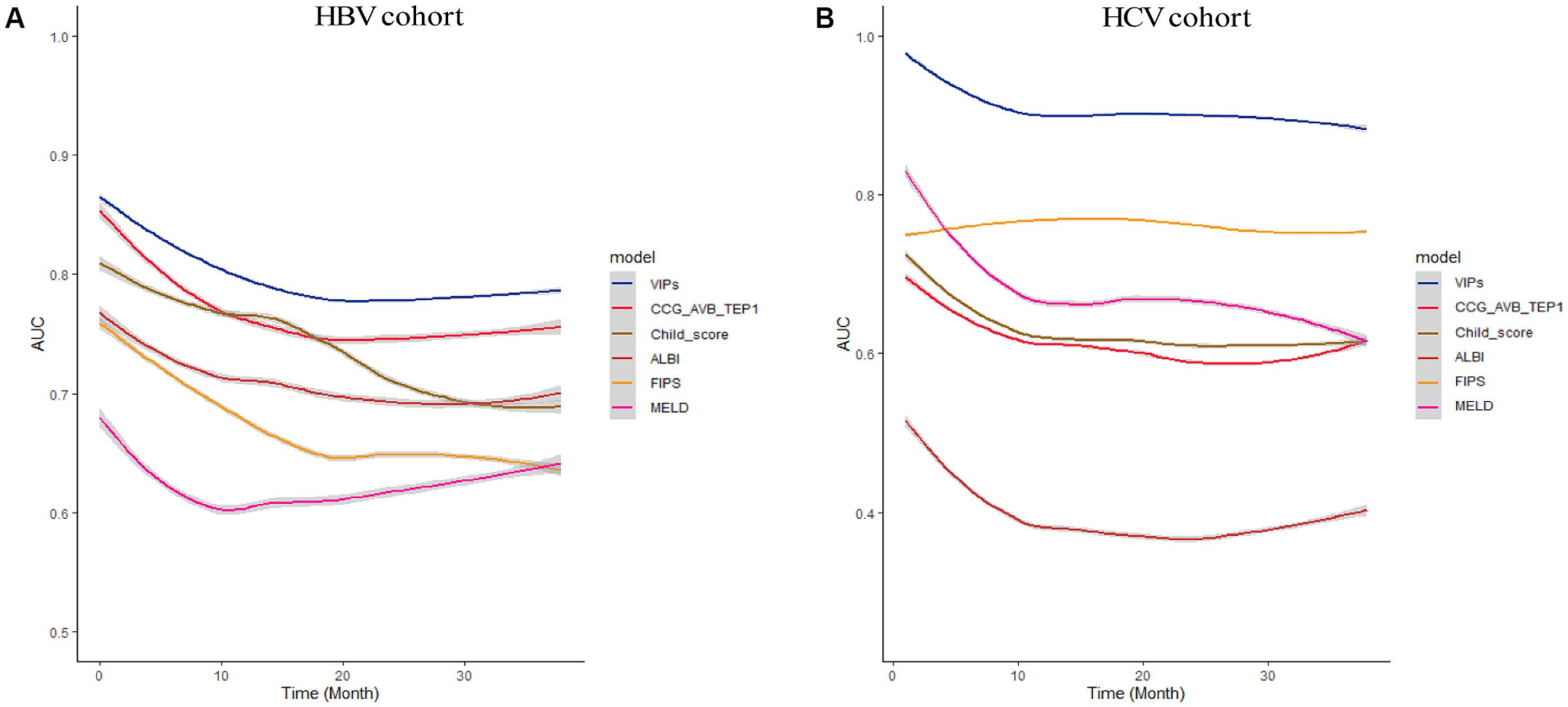
Subgroup analysis of HBV cohort and HCV cohort. The time-dependent area under the ROC curve (AUC) of the VIP score compared with other existing scores for 1- and 3-year survival prediction in the HBV cohort **(A)** and HCV cohort **(B)**.

In the external validation cohort, the VIP score also demonstrated excellent discriminative ability. This cohort had the largest C-index (0.869) compared to other scores (Table 2). The AUCs of the VIP score for 1- and 3-year survival prediction in the external validation cohort were 0.771 and 0.775, respectively (Figures 4C,D). DCA of the VIP score demonstrated a higher net benefit than the other scores in the external validation cohort (Figure 6B). Similarly, the VIP score had good calibration at 1 year and 3 years (Figures 5C,D). In subgroup analysis, the VIP score showed fair to excellent performance for 1- and 3-year survival prediction in the HBV and HCV cohorts, which was superior to other scores (Figures 7A,B).

### 3.5 Risk stratification based on the VIP score

**Figure 8:**
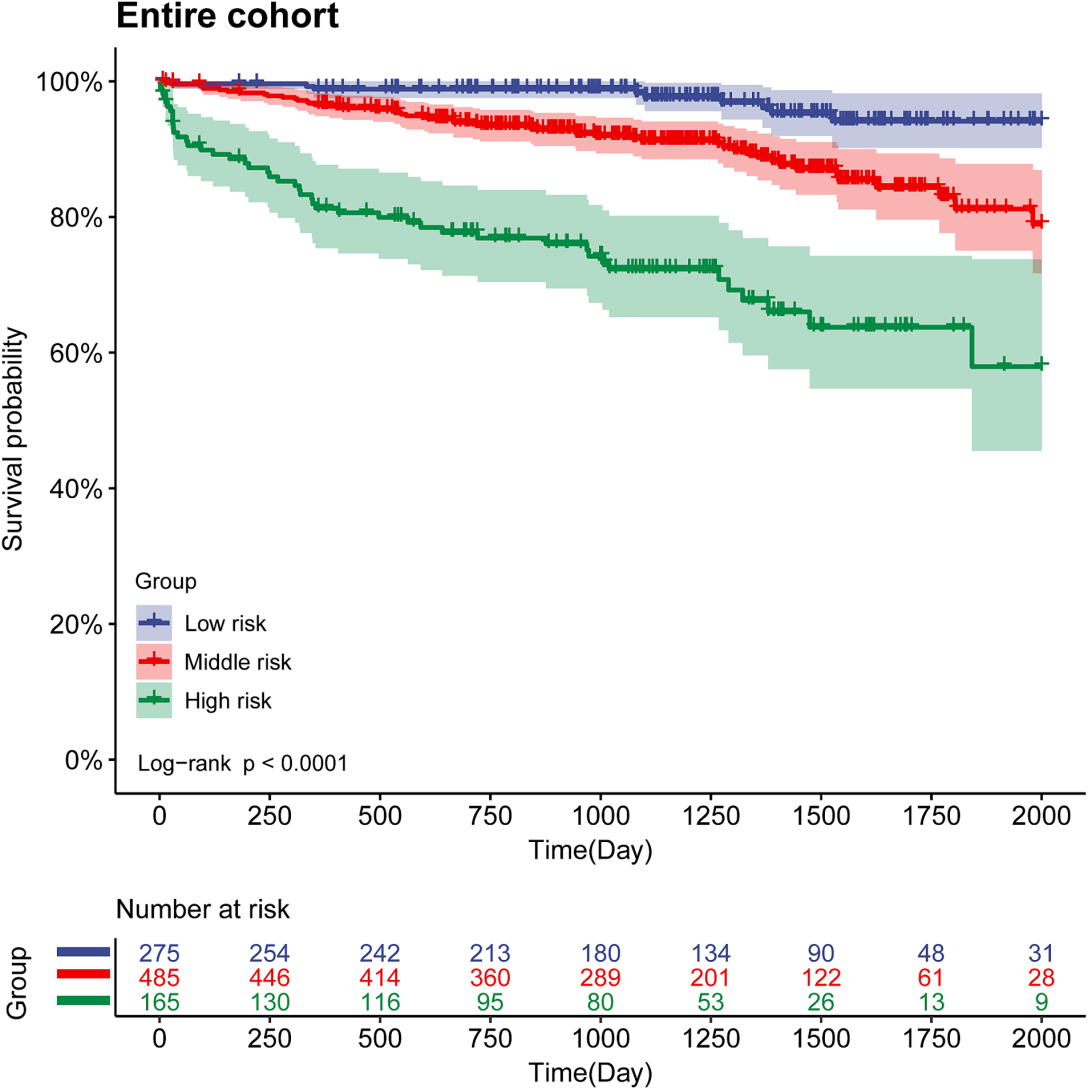
Overall survival risk stratification of the VIP score model in the entire cohort.

X-tile software generated two optimal cutoff values (83 and 115), which were used to divide the entire cohort into three risk groups: low risk (score < 83), medium risk (score 83–115), and high risk (score > 115). The 3-year cumulative mortality rates for the low-, medium-, and high-risk groups were estimated to be 34.5, 40.4, and 51.5% in the entire cohort, respectively. The Kaplan–Meier curve showed that, among the three risk groups, higher risk was associated with lower the overall survival (OS) (Figure 8, entire cohort, *χ*2 = 86.37, *p* < 0.001). Therefore, the VIP score could effectively stratify the risk of patients with viral hepatitis cirrhosis-related portal hypertension treated with TIPS placement.

## 4 Discussion

In China, the most common causes of liver cirrhosis are chronic HBV and HCV infections (21). Due to variations in healthcare resources and economic levels, the management of these patients differs from that in Western countries. Furthermore, liver transplants are relatively difficult to obtain in China. Therefore, TIPS is one of the major options for the treatment of complications associated with portal hypertension in cirrhosis. The prognosis of post-TIPS is usually based on the clinical characteristics of patients before TIPS placement. The non-invasive method is an effective and promising way to predict the prognosis after TIPS. However, there is no established scoring model focused on HBV and HCV patients to predict the survival of post-TIPS patients. In this study, we developed and externally validated a novel model to predict post-TIPS survival based on HBV and HCV Chinese patients. The performance of the model is good in terms of calibration and clinical benefit indicators.

In this multi-center retrospective study, we developed a simple score composed of six variables (age, ascites, albumin, PT, TBIL, and sodium) to predict the 1-year and 3-year survival of TIPS treatment for patients with viral hepatitis cirrhosis-related portal hypertension. According to the latest research, Han and Zhao conducted two models to predict post-TIPS prognosis based on Chinese cohorts. The CCG-AVB-TEP1 model proposed by Han (19) was based on non-TIPS cohorts (cirrhosis-related AVB patients who were treated with endoscopy plus drugs) and validated by a small sample of preemptive-TIPS cohorts. The model ^MT^ proposed by Zhao (22) did not analyze the etiology distinction. In addition, both models were used for predicting short-term survival after TIPS placement. Our study has several advantages: (1) The six variables were objective indicators that can be easily acquired. (2) This study was conducted based on the largest cohort of HBV and HCV-related cirrhosis patients who received TIPS implantation in China, which could make our results more representative and reliable. (3) The existing scores are mainly applicable to short-term prediction (time less than 1 year) after TIPS placement, while our developed score extends the prediction time to 3 years after TIPS and achieves good prediction performance. (4) There were major differences in economic levels and medical resources between the southwest and northeast in China. Our study was a multi-center effort that first developed a robust model based on the data from eight centers in the southwest of China. We then externally validated this model using data from a center in the northeast of China, where it also demonstrated good performance. This suggests that our model is both representative and consistently reliable across different Chinese cohorts.

This study first constructed a model including these six variables, especially for the prediction of post-TIPS prognosis in HBV and HCV-related cirrhosis patients. According to previous studies, HBV and HCV-related cirrhosis patients exhibit worse liver function and renal function and more serious portal hypertension (23–25). Therefore, these six variables might be more suitable for the prediction of post-TIPS prognosis in HBV and HCV-related cirrhosis patients. Although hepatic encephalopathy (HE) is a proven prognostic factor for TIPS, we did not take HE as a screening variable because the diagnosis of HE is mainly based on clinical manifestations according to the subjective experience of the doctor, which cannot be easily uniformed in each center. Moreover, minimal hepatic encephalopathy cannot be diagnosed in a timely manner. This change improved the stability and ability of our model compared to the classic CTP score. Interestingly, we found that creatinine was not an independent prognostic factor, which is why those scoring models containing creatinine, such as the MELD, CCG, and FIPS scores, proved to be suboptimal in assessing prognosis. This may be because esophageal variceal bleeding was the main indicator for TIPS in our study, while refractory ascites accounted for only 6.6% of the cases. Patients with variceal bleeding exhibited an earlier stage of cirrhosis than patients with refractory ascites. This is the reason why fewer patients had severe renal impairment in our study. In fact, creatinine is the most controversial scoring parameter because it has many influencing factors in patients with liver cirrhosis, such as age, sex, malnutrition, sarcopenia, and drugs. Furthermore, patients with non-liver-related renal dysfunction cannot be effectively identified. However, several studies have shown that renal function improves significantly after TIPS in patients with preoperative renal insufficiency. Hence, our study showed that creatinine is not an independent prognostic factor in patients undergoing TIPS implantation.

This study has several limitations. First, our exclusion criteria included patients with preoperative hepatocellular carcinoma, so our score is not applicable to these populations. Second, the sample size was calculated based on the univariate screening results, rather than all variables, which may overestimate the power. Third, preoperative parameters such as serum albumin, serum sodium level, and ascites volume are easily affected by treatment factors. However, insufficient data on patients’ preoperative treatment strategies make it challenging to calibrate the baseline parameters, which may affect the predictive performance of our model. Fourth, the result may not be generalized to other populations/ethnicities. Finally, our external validation cohort was relatively small. Additional sample sizes for validation are needed.

## 5 Conclusion

We developed and externally validated a prognostic score for TIPS placement based on the largest cohort of patients with viral hepatitis cirrhosis-related portal hypertension using variables that are easily accessible in clinical practice. This risk score can stratify patients into low-, medium-, and high-risk groups and predict their 1-year and 3-year survival rates after TIPS placement. This information helps patients and their families make informed decisions and enables doctors to develop personalized treatment strategies. Although our score performed well overall, it still needs to be validated in larger cohorts or higher-quality studies, which should be the focus of future studies.

## Data Availability

The raw data supporting the conclusions of this article will be made available by the authors, without undue reservation.
